# Preserved multisensory body representations in advanced age

**DOI:** 10.1038/s41598-019-39270-7

**Published:** 2019-02-25

**Authors:** Martin Riemer, Thomas Wolbers, Esther Kuehn

**Affiliations:** 1 Aging & Cognition Research Group, German Center for Neurodegenerative Diseases (DZNE), 39120 Magdeburg, Germany; 2 Center for Behavioral Brain Sciences (CBBS), 39118 Magdeburg, Germany

## Abstract

The internal representation of the body emerges via the integration of multisensory body cues. Sensory signal transfer and the ability to integrate multisensory information deteriorate significantly with increasing age. However, there is little empirical evidence on age-related changes in body representations based on multisensory integration. Here, we used a standard paradigm for evaluating body representations based on multisensory integration, the rubber hand illusion, and compared the amount of proprioceptive drift and changes in perceived body ownership triggered by the integration of visual, tactile, and proprioceptive cues between younger and older adults. To account for potential age-related differences in the temporal stability of the illusion, proprioceptive drift was measured at five different time points. Our results show that older adults used synchronous visuo-tactile cues similarly to younger adults to update both the position of their own hand, and their feeling of ownership over the artificial hand. Independent of visuo-tactile synchrony, older adults perceived their hand as closer to their body than younger adults did, and showed a less stable representation of this in-depth hand position. This proprioceptive bias towards the body did not correlate with the strength of the illusion. Our results indicate that the integration of visual and tactile cues is largely preserved in advanced age when used to update limb position, whereas proprioception worsens with age. This may be linked to two different pathways that underlie changes in body representations over the life span.

## Introduction

In contrast to intuition, the sense of the bodily self is not a static construct but dynamically emerges from the integration of multisensory cues^1–3^. In fact, body representations are created ‘on the fly’ and can be altered within short time periods: Bodily illusions such as the rubber hand illusion (RHI)^4^, or the Pinocchio illusion^5^ impressively demonstrate that even short periods of multisensory integration lead to a re-construction of body representations, quantified as proprioceptive drift of one’s own hand towards an artificial hand, in the case of the RHI^3,6^, or as changes in the perceived size or shape of body parts, in the case of the Pinocchio illusion^5,7^.

Changes in body representations are especially relevant in the context of aging. Sensory modalities deteriorate with age, and body representations are updated less efficiently in older compared to younger adults^8–12^. Those factors are assumed to cause maladaptive behavior, such as an increasing number of falls, or decreased manual dexterity. Based on a review of the available literature on changes in embodiment in older age, Kuehn *et al*.^11^ recently concluded that specifically the degradation of sensory stimulus perception, changes in multisensory integration, and changes in temporal updating in older compared to younger adults have far-reaching consequences on a multitude of sensory, motor, and cognitive functions^11^. However, empirical studies specifically investigating age-related changes in body representations are scarce.

The RHI is a standard tool to measure changes in perceived body ownership and body posture, and has increased our understanding of body representations in a multitude of clinical conditions, such as in limb amputees^13^, in people with autism^14^, schizophrenia^15^, and eating disorders^16^. The RHI has also been used to study multisensory integration in children^17^. When inducing the RHI, an artificial hand is placed visibly at an anatomically plausible position in front of the participant, while the participant’s own hand is hidden from view. The experimenter then applies tactile stimuli synchronously to the participant’s own (hidden) hand, and to the (visible) artificial hand. In this situation, the integration of visual, tactile and proprioceptive cues induces a perceptual shift of the participants’ own hand towards the location of the artificial hand. As a control condition, tactile and visual stimuli are applied asynchronously. Proprioceptive drift is then defined as the difference in perceived location between the synchronous and the asynchronous condition and serves as a marker for the specific changes associated with co-occurring multisensory cues.

One previous study used the RHI paradigm to characterize changes in body representations over the life span^17^. The authors showed similar proprioceptive drifts in children (4 to 9 years old) and adults (mean age 23.9 years) towards the artificial hand in the synchronous compared to the asynchronous condition^17^, and concluded that the visual-tactile pathway that is used to update limb position is already functional in young children. This is surprising, as other studies suggest that multisensory integration mechanisms undergo further development at an age of 10 to 11 years^18–21^. Moreover, older adults – in most studies defined as at least over 60 years of age – exhibit a different weighting of sensory cues compared to younger adults, and have partly reduced abilities to combine multisensory cues in an optimal way^22–24^. In addition, vibration-induced limb movement illusions are significantly fewer, less salient, and delayed in older adults compared to younger adults^25^. This may be accompanied by a less stable updating of limb position in advanced age, but may also lead to increased integration of visual and tactile cues to compensate for worsened proprioceptive abilities^25^.

Only one previous study has investigated age-related changes in the RHI in younger and older adults^26^. The authors did not find significant differences in perceived limb position in older adults (61 to 80 years old) compared to younger adults (20 to 35 years old) when tactile and visual stimuli were applied synchronously. However, because there was no asynchronous control condition, specific changes in body representations based on multisensory integration were not investigated in this study.

Here, we targeted three specific questions: First, we tested whether the proprioceptive drift, defined as the difference value between a synchronous and an asynchronous stimulation condition, differs between younger and older adults (H1: Age-related changes in implicit body representation). Second, we investigated whether this condition also induces changes in the conscious perception of body ownership by asking participants about their subjective body experience during the induction phase (H2: Age-related changes in explicit body representation). Finally, we investigated whether there are differences in the temporal stability of the RHI in older compared to younger adults (H3: Age-related changes in temporal stability of the illusion). Answering these questions allows gaining novel insights about age-related changes in body representation and body ownership, which are assumed to influence a multitude of functional differences in advanced age.

## Methods

### Participants

**Figure 1:**
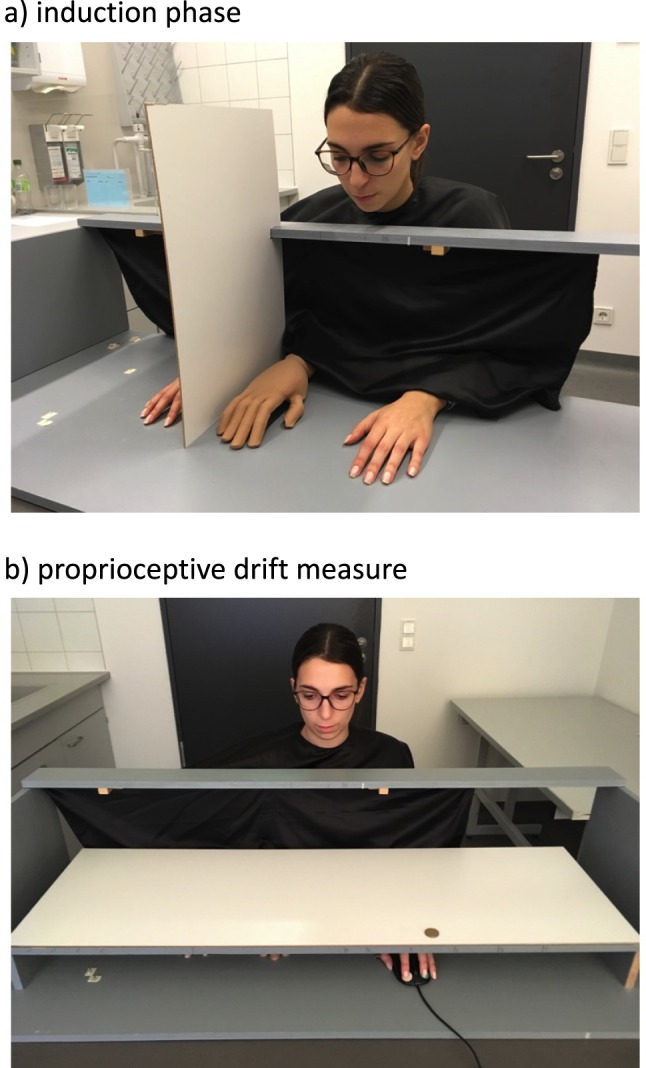
Depiction of the experimental set-up. During the induction phase (**a**), brush strokes were applied to the artificial hand (visible) and to the participant’s own right hand (hidden from view by an occluding screen). To assess proprioceptive drift (**b**), a white board was placed horizontally above the hands. From the ceiling, a beamer projected a white screen on the board. Participants indicated the perceived location of their own right index fingertip by moving the mouse curser above the respective location. Each of five subsequent responses started from the same starting point (located the upper edge of the board).

Twenty younger (7 males, mean age 23.3 years, ranging from 18 to 30 years) and twenty older participants (7 males, mean age 70.8 years, ranging from 65 to 79 years) were recruited from the local community in Magdeburg, Germany. Exclusion criteria for taking part were deficits in sensory processing, abnormal motor behavior, professional sportsmen/-women, current illness (including neurological disorders), and a history of mental disorders. All participants were right-handed, received monetary compensation for their participation, and gave written informed consent to the experimental protocol, which was approved by the ethics committee of the Otto-von-Guericke-University of Magdeburg, Germany. All methods were performed in accordance with the relevant guidelines and regulations. One participant gave informed consent for the online publication of identifying images (Fig. 1).

### Experimental set-up

Participants sat at a desk and placed their hands into a wooden framework (125 cm × 50 cm × 25 cm), as depicted in Fig. 1a. An artificial right wooden hand was placed 15 cm to the left of their own right hand (measured from the tip of the index finger), which was hidden from view by an occluding screen. Participants were instructed to adjust their body midline halfway between the artificial right hand and their own left hand, which was placed 31 cm to the left of the artificial hand. This experimental set-up has been used in a number of previous studies on the RHI^27–30^.

To allow for a direct comparison between age groups, an age-neutral artificial hand wearing an elastic, skin-colored glove was used for both younger and older participants. Participants themselves did not wear gloves. Before the experiment started, we measured the size of the participants’ own right hands and asked them to rate the subjectively perceived similarity between the artificial and their own hand on a 10-point Likert scale.

### Experimental procedure

The (visible) artificial and the participants’ (hidden) own right hand were stroked by the experimenter with two soft paintbrushes, either synchronously (illusion condition) or asynchronously (control condition). Within each age group, the order of stroking condition was randomized across subjects. Strokes were applied to the right index fingers of both hands with a mean frequency of 0.2 Hz (approximately every five seconds). Each stroke started from the first phalanx to the fingernail in a smooth and continuous movement, lasting for about one second. In the asynchronous condition, the temporal delay between stroke onsets at the own and the artificial hand was approximately two seconds. The stroking procedure was guided by acoustic signals presented to the experimenter via headphones.

After the induction phase, participants were instructed to close their eyes. The experimenter removed the occluding screen and placed a wooden board with a white surface horizontally above the participants’ hands (Fig. 1b). A beamer (Acer P1163, fixed 1.85 m above the desk) projected a white screen (81.1 × 46.6 cm^2^) on the board’s surface. Participants then opened their eyes and used a computer mouse – controlled by their left hand – to drag crosshairs above the two-dimensional surface of the board. They were instructed to position the crosshairs above the location where they perceived the tip of their own right index finger, and to confirm this location by mouse click. This procedure was repeated five times. Before each new response, participants had to move the crosshairs to a start location at the upper-left corner of the projection area. This ensured novel positioning of the fixation cross for each of the five responses. Compared to classic pointing movements with the contralateral hand^6,29^, this technique has the advantage that only minimal motion with the non-stimulated hand is required, thereby reducing the influence of bodily motion on the stability of the RHI^6^, and also reducing bilateral integration of proprioceptive information^31^.

After the assessment of proprioceptive drift, participants completed an RHI questionnaire, consisting of nine statements regarding the subjective experience of the illusion during the induction phase. Agreement with these statements was indicated on a Likert scale involving seven steps (from −3 = “strongly disagree” to 3 = “strongly agree”). Questionnaire items were selected from Longo *et al*.^32^ and are listed in Table 1. According to Longo *et al*.^32^, items 1, 4 and 7 refer to the embodiment of the artificial hand, items 2, 5 and 8 to the affective valence of the experience, and items 3, 6 and 9 served as control questions. Affective valence was assessed to exclude the possibility that age-related differences in the RHI experience can be explained by the pleasantness of tactile sensations, which can change during the lifespan^33,34^.

**Table 1:** Questionnaire items and categories.

item	item category
1. It felt as if the artificial hand was my own hand.	ownership
2. I found that experience enjoyable.	affect
3. My right hand felt numb.	control
4. The artificial hand seemed to resemble my own right hand (in terms of shape, skin structure etc.).	ownership
5. The touch on my right finger was pleasant.	affect
6. It seemed as if I had two right hands.	control
7. It seemed as if the artificial hand was part of my body.	ownership
8. I found that experience interesting.	affect
9. It seemed as if I couldn’t really tell where my right hand is.	control

In summary, each of the two stroking conditions (synchronous vs. asynchronous) involved an induction phase (2 min), five responses to indicate the perceived position of the own right index fingertip (approx. 1 min), and the completion of an RHI questionnaire (approx. 1 min).

### Statistical analysis

Proprioceptive drift was quantified by the difference between the indicated and the actual location of the own right index fingertip, measured laterally (left - right), and in-depth (towards the body – away from the body). Positive values indicate a leftward drift towards the artificial hand or a backward drift towards the body, respectively.

Data on proprioceptive drift were analysed separately for the lateral and the in-depth axes, according to linear mixed effects models (2 × 2 × 5 factorial design), including the between-subjects factor age group (old vs. young) and the within-subjects factors synchrony (synchronous vs. asynchronous) and trial (five sequential responses). Subjects were included as random factor. Precision of pointing responses was calculated by the standard deviation of the five sequential responses and analyzed by means of t-tests. Assumptions of normality were tested by means of Shapiro-Wilks tests.

As normality cannot be assumed for Likert scale data, RHI questionnaire items and the similarity ratings (between own and artificial hand) were analysed with non-parametric Wilcoxon signed-rank tests for paired samples (two-tailed). Correlation analyses (one-tailed) were based on the difference values between synchronous and asynchronous conditions.

Statistical analysis was conducted using R (version 3.0.2).

## Results

We first assured that our results cannot be accounted for by differences in physical body properties between younger and older adults. Neither hand size (t_35.9_ = 0.6, p > 0.5) nor the perceived similarity between participants’ own hand and the artificial hand (W = 192.5, p > 0.5) differed between younger and older participants.

**Figure 2:**
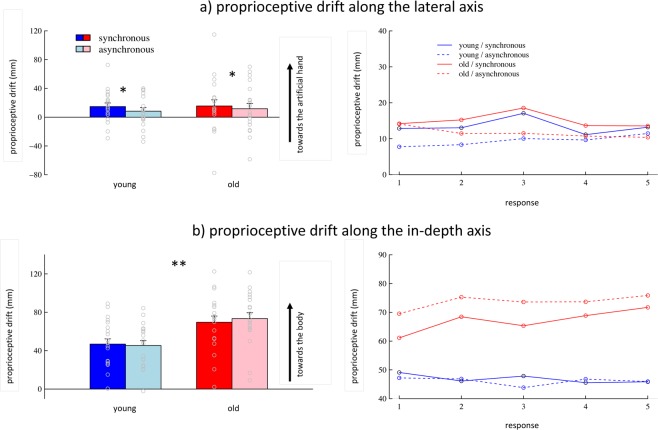
Proprioceptive drift for young (blue) and old (red) participants (**a**) along the lateral axis, i.e., towards the artificial hand, and (**b**) along the in-depth axis, i.e., towards the own body. Right graphs show all five subsequent responses individually. Error bars show standard error across subjects (**p < 0.01; *p < 0.05).

As expected, analysis of proprioceptive drift along the lateral axis revealed a significant main effect of synchrony across age groups, and time points (F_1,352_ = 5.9, p = 0.02). After synchronous stroking, perceived hand location shifted towards the artificial hand, demonstrating a general effect of the illusion condition on proprioceptive drift. Neither the main effect of trial (F_1,352_ < 0.1, p > 0.5) nor the main effect of age group (F_1,38_ < 0.1, p > 0.5) reached a significant level. Furthermore, there were no interaction effects (synchrony × trial: F_1,352_ < 0.1, p > 0.5; synchrony × group: F_1,352_ < 0.1, p > 0.5; trial × group: F_1,352_ = 0.8, p = 0.38; synchrony × trial × group: F_1,352_ = 0.5, p = 0.46). Thus, while there was an effect of synchrony on proprioceptive drift along the lateral axis, this effect was not different between age groups. Normality regarding the lateral proprioceptive drift can be assumed for both age groups and both synchrony conditions (all p > 0.16). The results for proprioceptive drift are depicted in Fig. 2.

A different pattern of results emerged for proprioceptive drift along the in-depth axis. While the main effect of synchrony was only marginally significant (F_1,352_ = 3.9, p = 0.05), there was an interaction between synchrony and age group (F_1,352_ = 7.7, p = 0.006). After synchronous compared to asynchronous stroking, perceived hand position drifted away from the body in older, but not in younger participants. However, separate t-tests for both age groups did not reveal a significant effect on a group level, neither for younger (t_19_ = 0.6, p > 0.5) nor for older adults (t_19_ = −0.7, p = 0.25). Furthermore, we found a main effect of age group on proprioceptive drift along the in-depth axis (F_1,38_ = 9.8, p = 0.003), indicating that older adults perceived their hand as being located closer to their body than younger adults did, irrespective of stroking condition (M_young_ = 46 mm, M_old_ = 71 mm). The main effect of trial was not significant (F_1,352_ = 1.3, p = 0.26), but there was an interaction between trial and age group (F_1,352_ = 3.9, p = 0.04): While the perceived hand position after the induction phase remained stable in younger adults, it slowly drifted towards the body in older adults. All other interaction effects were non-significant (synchrony × trial: F_1,352_ < 0.1, p > 0.5; synchrony × trial × group: F_1,352_ = 0.5, p = 0.47). Normality regarding the in-depth proprioceptive drift can be assumed for both age groups and both synchrony conditions (all p > 0.26).

The correlation analysis revealed that the synchrony-independent proprioceptive misperception of the own hand towards the body, which was specifically observed in older adults, was not associated with the extent of proprioceptive drift along the lateral axis induced by synchronous visuo-tactile stimulation (t_18_ = 0.4, p = 0.35).

We also compared the precision of pointing responses between younger and older participants. No significant differences were found, neither along the lateral axis (synchronous condition: t_28.8_ = 0.9, p = 0.18; asynchronous condition: t_32.9_ = 0.7, p = 0.24) nor along the in-depth axis (synchronous condition: t_25.2_ = 1.2, p = 0.12; asynchronous condition: t_32.2_ = 0.6, p = 0.28).

**Figure 3:**
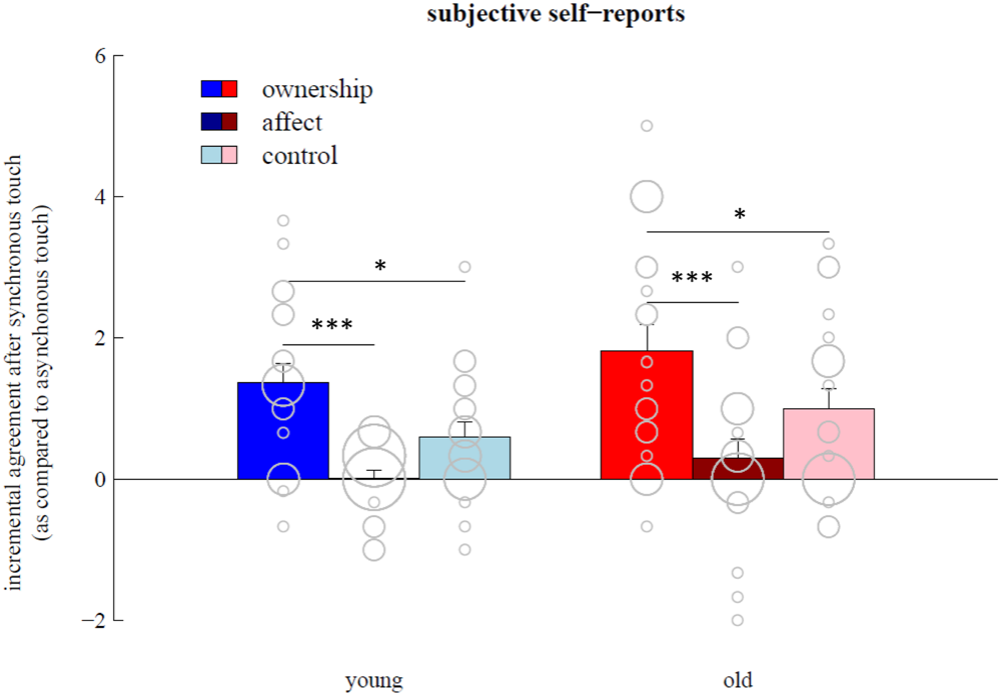
Synchrony-induced changes in perceived ownership over the artificial hand (blue and red bars) are significantly larger than changes in the affective state experienced during the illusion (dark colours) and changes in responses to control items (light colours), both for young and old participants. Grey circles indicate individual data points. Error bars show standard error across subjects (***p < 0.001; *p < 0.05).

On the phenomenological level, there was no significant difference with respect to how younger and older adults experienced the illusion (Fig. 3). Perceived ownership over the artificial hand, quantified by the mean of three ownership-related items (items 1, 4 and 7 in Table 1) was significantly modulated by the synchrony of stimulation in younger (V = 149.5, p < 0.001) as well as in older participants (V = 149, p < 0.001). In both age groups, the effect of synchrony on ownership-related items was significantly larger than for affect-related items (young: V = 202, p < 0.001; old: V = 174, p < 0.001), and for control items (young: V = 96.5, p = 0.02; old: V = 120, p = 0.02). For all three item categories, the effect of synchrony did not significantly differ between younger and older participants (ownership: V = 71, p = 0.34; affect: V = 69, p = 0.30; control: V = 75, p > 0.5).

Synchrony-induced changes in proprioceptive drift were not correlated with corresponding changes in ownership ratings (t_38_ = −0.6, p > 0.5).

## Discussion

We used the rubber hand illusion (RHI)^4^, a standard paradigm to investigate changes in body representations driven by multisensory integration, and asked whether the ability to estimate the position of one’s own hand based on the integration of visual and tactile information differs between younger and older adults. We had a two-sided hypothesis on whether or not there would be age-related differences in implicit and explicit markers of the RHI, with current literature supporting both possible outcomes (increased integration versus deteriorated sensory input and less optimal weighting of integrated sensory information). Our results show no significant differences in implicit and explicit markers of the RHI between younger and older adults. The lateral drift of participants’ own hands towards the artificial hand, an established marker to measure the RHI, did not significantly differ between younger and older adults. There was also no significant difference in subjective reports of perceived ownership over the artificial hand; both groups felt increasing ownership over the artificial hand after the synchronous compared to the asynchronous stroking condition. The proprioceptive drift along the lateral axis was also stable over time points in both age groups, and did not start later, or diminish earlier in older compared to younger adults. This provides evidence towards a preserved pathway to integrate tactile, proprioceptive, and visual cues to update body representations in older age.

We also found a significantly increased proprioceptive bias in older compared to younger adults: Older adults perceived their hands as significantly closer to their own body than younger adults did, irrespective of the tactile stimulation condition (i.e., synchronous vs. asynchronous). This in-depth bias did not correlate with the proprioceptive drift as induced by the RHI. This main effect of age group was accompanied by an interaction effect between age group and synchrony for the in-depth bias. However, an effect of synchrony on the proprioceptive drift in-depth was not confirmed within single groups. In addition, we found that older adults showed a less stable position sense over repeated responses in the in-depth axis: The hand slowly drifted towards the body with subsequent responses. This effect, however, was independent of the stimulation condition (synchronous vs. asynchronous), and hence not triggered by the temporal integration of visual and tactile cues during synchronous stimulation.

Our results are in line with the findings of Cowie *et al*.^17^, who showed that children aged 4–9 years do not show differences in the emergence of the RHI compared to adults, but that they differ in basic proprioceptive abilities in a non-illusion condition (see also^35^ on this point). In the study by Cowie *et al*.^17^, basic abilities to estimate the position of the hand based on proprioceptive information was diminished as a function of (younger) age, whereas the lateral drift induced by synchronous stroking was not influenced by age. Nava *et al*.^21^ also found that the somatic rubber hand illusion has similar effects on estimated hand position in 8–9 years old children and in adults. Here, however, the illusion could not be elicited in very young children aged 4–5 years. Finally, Palomo *et al*.^26^ also provided evidence that the RHI has similar effects on perceived hand position in younger and older adults. These results together with our results favour the view that the neuronal pathway that allows the rapid integration of visual and tactile information to form an online representation of the bodily self is both early maturing (i.e., already present in children) *and* stable over the course of our lives (i.e., preserved in older adults), whereas the pathway that mediates proprioceptive abilities may develop later, *and* degrade earlier (see also^36^). It is important to note, however, that only an experiment where proprioceptive acuity is measured in the absence of tactile stimulation can confirm this hypothesis.

Several studies reported differences between the perception of lateral distances and distances in-depth^37–39^, an effect that has been shown to change with increasing age^40^. For example, there are differences in proprioceptive lateral bias in expert dancers compared to non-dancer controls, likely driven by less bilateral integration of proprioceptive information in expert dancers^32^. Position judgments along the lateral axis are expected to rely more on vision and less on proprioception, whereas position judgments in-depth are expected to rely more on proprioception and less on vision^39^. The increased in-depth bias in older compared to younger adults found here favours the view that particularly the proprioceptive sense deteriorates with age due to both peripheral and central differences in the proprioceptive system. The updating of proprioceptive information based on multisensory cues, however, remains stable over time, and allows the flexible updating of body representations.

This is also in line with a recent study by Chancel *et al*.^25^. These authors induced limb movement illusions based on either proprioceptive, visual, or tactile cues. They found preserved or even enhanced illusion effects in older adults when visual or tactile information was used to induce the illusion. This was interpreted as enhanced integration of multisensory cues, perhaps to compensate for the degraded proprioceptive abilities in older adults that we also observe here. In a broader context, this indicates that visuo-tactile feedback devices could serve as a valuable tool in older adults to overcome difficulties in basic proprioception or motor control, which are, for example, associated with an increased risk of falls^41,42^. A practical implication of this may be the development of functional textures on floor surfaces in nursing homes^43,44^, or the use of tactile feedback devices during walking or when learning new skills.

Another interesting aspect about the preserved sense of body representations in older adults that we show here, measured by proprioceptive updating based on visual and tactile cues, is the stability of the lateral proprioceptive drift over time. A large area of research indicates that neuronal representations, but also cognitive phenomena such as attention are less stable over time in older compared to younger adults^45,46^. Therefore, it could be expected that the proprioceptive drift induced by the illusion may be less stable over time in older compared to younger adults, i.e., that the effect of multisensory stimulation on changes in body representation would be more affected by temporal fluctuations, and disappear earlier. However, we did not find such a modulation. The multisensory effect remained similarly stable in older and younger adults, at least over a time frame of around 1 minute (see also^3^). Because basic proprioceptive position sense of the hand along the in-depth axis (irrespective of stroking condition) in fact did show worsening over time in older adults compared to younger adults, it seems that the multisensory percepts but not basic proprioceptive abilities are stable in older adults.

Taken together, our results show that the RHI is functional in older age and that visuo-tactile integration mechanisms that are used to construct and update body representations are not significantly degraded in older adults. This is interesting, because a degraded representation of the bodily self is usually assumed in older adults^11^. Our results may inspire future research on the preserved sense of self in older compared to younger adults.
